# A Multidimensional Model of Public Health Approaches Against COVID-19

**DOI:** 10.3390/ijerph17113780

**Published:** 2020-05-26

**Authors:** Mehrab Nazir, Iftikhar Hussain, Jian Tian, Sabahat Akram, Sidney Mangenda Tshiaba, Shahrukh Mushtaq, Muhammad Afzal Shad

**Affiliations:** 1 School of Economics and Management, Jiangsu University of Science & Technology, Zhenjiang 212003, China; mehrabnazir9@gmail.com (M.N.); christhackman@outlook.com (S.M.T.); 2 Dean, Faculty of Computing & Engineering, University of Kotli Azad Jammu & Kashmir, Kotli 11100, Pakistan; 3 Department of Econmomics, University of Kotli Azad Jammu & Kashmir, Kotli 11100, Pakistan; sabahat_abrar@hotmail.com; 4 Department of Business Administration, University of Kotli Azad Jammu & Kashmir, Kotli 11100, Pakistan; shahrukh.dbms@must.edu.pk; 5 Department of Commerce, University of Kotli Azad Jammu & Kashmir, Kotli 11100, Pakistan; afzaalshaad@gmail.com

**Keywords:** COVID-19, social media, preventive behavior, social actors, pandemic

## Abstract

COVID-19 is appearing as one of the most fetal disease of the world’s history and has caused a global health emergency. Therefore, this study was designed with the aim to address the issue of public response against COVID-19. The literature lacks studies on social aspects of COVID-19. Therefore, the current study is an attempt to investigate its social aspects and suggest a theoretical structural equation model to examine the associations between social media exposure, awareness, and information exchange and preventive behavior and to determine the indirect as well as direct impact of social media exposure on preventive behavior from the viewpoints of awareness and information exchange. The current empirical investigation was held in Pakistan, and the collected survey data from 500 respondents through social media tools were utilized to examine the associations between studied variables as stated in the anticipated study model. The findings of the study indicate that social media exposure has no significant and direct effect on preventive behavior. Social media exposure influences preventive behavior indirectly through awareness and information exchange. In addition, awareness and information exchange have significant and direct effects on preventive behavior. Findings are valuable for health administrators, governments, policymakers, and social scientists, specifically for individuals whose situations are like those in Pakistan. This research validates how social media exposure indirectly effects preventive behavior concerning COVID-19 and explains the paths of effect through awareness or information exchange. To the best of our knowledge, there is no work at present that covers this gap, for this reason the authors propose a new model. The conceptual model offers valuable information for policymakers and practitioners to enhance preventive behavior through the adoption of appropriate awareness strategies and information exchange and social media strategies.

## 1. Introduction

Several patients with symptoms of pneumonia of unknown facts were reported in mid of December, 2019 in Wuhan, Hubei province, China [1]. After an investigation by World Health Organization (WHO), it was identified as a new virus called COVID-19, and with time, it spread rapidly throughout China and other countries [2]. According to WHO, it has been reported that there are 2.6 million confirmed cases, 0.184 million deaths, and 0.722 million recoveries from 2019-nCoV worldwide. As evidence of this spread, one such case was reported on January the 24th, 2020 when a person reportedly from China came to Pakistan on 21st of January 2020 via Dubai. On the 24th he was examined and was found to be positive for COVID-19. It was then that Pakistan for the first time was exposed to the virus and became part of the affected countries globally. According to the government of Pakistan’s reports, today the number of confirmed cases in Pakistan is 27,474 and the number of confirmed deaths is 618. Punjab and Sindh are the two mainly affected provinces. Moreover, due to the lack of medical resources in developing nations, Pakistan also faces challenges in preventing the spread of re-emerging and new infectious diseases. Thus, during the outbreak of infectious disease, these countries focus on alternatives to medical facilities to overcome its spread. Awareness and accurate information bring the behavioral changes among the people; they can be perceived as half treatment without any expense. 

Social media has become an important source to broadcast awareness and information regarding control of infectious disease [3]. According to [4], social media consists of different applications, including social networking sites, and blogs, that are founded on the scientific and ideological foundation of web 2.0 (for example, Facebook, YouTube, and Twitter) that allow users to make, share content, and participate in different activities. Social media itself is a catch-all expression for sites that may consist of various social actions. Social media is designed of an electronic-mediated platform relying upon web-based innovations that permit users to make a profile and share ideas, imagines/clips, and information in the virtual networks system. 

Even though many cases which were initially reported exposed the seafood market in Wuhan, according to the current epidemiologic information, this virus is spreading from one individual to another at a very high rate of transfer [5]. With time, 2019-nCoV has infected almost all countries on the planet. Iafusco, Ingenito [6] argued that in these critical circumstances, it has been very difficult for developing nations to communicate with uninfected individuals along with infected persons because this virus can spread quietly from one person to another. It has become complicated for the governments and doctors to communicate with their citizens during an infectious disease outbreak, therefore, social networking sites are playing a critical role in enabling the populations to connect virtually. Some years ago, several disease outbreaks of the same nature, for example, Ebola, Zika flu, and Dengue fever, around the globe revealed insights into the significant power of communication strategies concerning such diseases [7].

Researchers said that social networking sites (SNS) are a scoping evaluation of the utilization of search queries in disease surveillance [8]. First reported in 2006, the viewed literature highlighted accuracy, speed, and cost performance that was comparable to existing disease surveillance systems and recommended the use of social media programs to handle all circumstances of infectious disease systems. Now, due to the advanced innovation of web 2.0, SNS have appeared to play an essential role for public health specialists to control the spread of such infectious disease. It has been observed that social networks perform an excellent evaluation of real-time data reporting which keeps the state and people posted for the possible solutions of public health safety during epidemics [9]. 

With this recognizable increase in infectious outbreak in Pakistan, public health centers are facing severe problems and challenges at work to act for the prevention of disease at various levels. Due to a lack of time and resources, it has become complicated for the nations to address these issues and challenges in a short time. A fear of physical spread hinders health sector workers to interact with patients and suspects in person. Therefore, the response time of governments and health departments to tackle the sign and symptoms which lead to the detection of both infectious and noninfectious diseases and their preventions, respectively, is affected by the updates and real-time reporting of social media. In this study, the researchers determined the outcome of social media on the preventive behavior among people about COVID-19, how individuals gain information and awareness knowledge through social media to control COVID-19. The study also analyzed the direct effect of social determinants on the preventive behavior of such conditions. The study was structured into five sections. After the introduction, the researches present thorough and critical analysis of current and most relevant literature along with hypotheses development. The third section contains material and methods used in this study to achieve the stated study objectives. The next section is about the main results of the study and discussions related to these results. The final section is about the main conclusions, findings, and the future research options.

## 2. Literature Review

### 2.1. SNSs As a Communication Tool for the Prevention of COVID-19 

Undeniably, online communication is used as an outlet for individuals to freely make and post data that is dispersed and extended worldwide after the advanced foundation of web 2.0. News media, conventional scientific outlets, and online networking sites create a platform for minority perspectives and individuals who are sometime, not being captured by other sources. Individuals seek information from an assortment of sources and continually update it from the health sector. Conventional news media has become recognizable as a comprehensive source of health information to prevent infectious disease in the public health sector. They provide information and awareness widely through social networking sites for reducing infectious diseases [10]. 

A few years ago, people did not have any communicational access to exchange their information directly with the government and health sectors, at that time, traditional media, such as newspaper, television, and so forth, played a critical role as a source of information exchange to the public [11,12]. Traditional media provided information about the disease to the public regarding public health issues [13]. Therefore, citizens relied on traditional media as a foundation of knowledge which helped them to understand the critical condition of risk and receive precautions about the issues. 

However, after the advanced foundation of web 2.0, there became a rapid change in the use of media technology, and people have increased their social media usage by registering in almost all the social network accounts, for example, Facebook, Twitter, and YouTube. This can also be seen from the increasing number of registered subscribers on all social web services to exchange their health information during any infectious disease outbreak [14]. Unlike traditional media, which just engaged users to a limited amount of used and obtained information, in social media people make their profile and share health-related information to others, also making comments on health-related posts, and these sites also give the users the opportunity to join any public health-related groups [15]. For example, at the time of the H1N1 flu virus outbreak, people used social networking sites as an information exchange medium and gave opinions related to health [16,17]. 

However, with the fast use of social networking sites, information access has changed, now people do not rely exclusively on the traditional or government news media, instead they trust SNS to get essential information from the public health sector. For example, Twitter was primarily utilized by the public for the exchange of experience, opinion, and knowledge among individuals during infectious disease [11]. Specifically, SNS have become a common source for general society to interchange their information when conventional news media offer very restricted information about an infectious disease outbreak due to some official pressure and limitations [18]. As per media policies, the public’s reliance on media will, in general, escalate at the time of significant emergencies. When information is not promptly accessible from traditional news media, people, as information makers and disseminators themselves, assemble electronic methods such as social media for information exchange [19]. 

Digital observation is an internet-based observation system that provides a current situation of public health problems by evaluating data stored digitally [20]. There are now numerous infectious disease observations in an epidemiological practice by which the predominance, outbreak, and extent of infectious disease are checked to build up patterns of active actions and advancements for management and control systems. The fundamental role of infectious disease observation is to observe, forecast, and reduce the harm caused by outbreak and epidemics situation as well as enhance the information system for specialists and the population concerning factors which could possibly be used in such conditions [21]. Revealing occurrences of outbreaks has been shifted from manual record-keeping systems to worldwide online communication networks through SNS [22]. Therefore, we can draw our first hypothesis as the following:

**Hypothesis** **1** **(H1).** *There is a significant relationship between social media and preventive behavior among people about COVID-19*.

### 2.2. Information Exchange as Mediator 

It is critical for public health sectors and government agencies to take any effective initiatives for the control of diseases, however, it is very difficult for developing countries to detect the infectious disease outbreak. Observational capacity for detection of infectious diseases could be very costly and the developing countries lack resources to measure the outbreak of infectious disease at the time of exposure. Hence, some social networking websites provide solutions to handle some of these challenges during an outbreak. Online networking sites provide a source of information to detect infectious outbreaks earlier with very cheap cost and provide a way to increase their reporting clearly [23]. 

The exchange of health information on social networking sites has been seen as an opportunity for health sectors to increase public health observation [24] and to predict and control infectious diseases [25]. Due to insufficient medical services in developing nations like Pakistan, the authorities face severe complications to contain and eradicate the chances of spread of such infectious diseases. Consequently, in case of an emergency, such communities start practicing alternatives to medical facilities to control the spread. Therefore, we can safely propose the following:

**Hypothesis** **2** **(H2).** *There is a significant relationship between social media exposure and information exchange about COVID-19 among people*.

**Hypothesis** **3** **(H3).** *Information exchange mediates the relationship between social media exposure and preventive behavior among people about COVID-19*.

### 2.3. Awareness Knowledge as Mediator 

Awareness regarding control of the infectious disease can overcome the financial burden for precautions. Earlier knowledge about the outbreak of disease can overcome the level of its spread [26]. Several methods can be used, like social media, internet access, TV, and so forth, by the nations to spread awareness about the precautions of disease. At present, mostly social networking platforms are being used as an important source to spread awareness of emergency to control an epidemic [27]. In the past, some researches have been conducted to evaluate the effect of social media to minimize the spread of infectious disease through preventive behavior. The results prove that social media is playing an essential role in overcoming the prevalence through prevention and reducing infection spread by awareness [28]. Awareness brings behavioral changes among communities. As the phrase states, “Prevention is better than cure”. Such awareness may be considered as half treatment without any expense. Therefore, the researcher draws their next hypotheses as the following:

**Hypothesis** **4** **(H4).** *There is a significant relationship between social media exposure and awareness knowledge*.

**Hypothesis** **5** **(H5).** *Awareness knowledge mediates the relationship between social media exposure and preventive behavior among people about COVID-19*.

### 2.4. Social Determinants As a Control Variable

Many researchers have proved that during the infectious outbreak, socio-economic factors profoundly influence the prevention behavior towards diseases. The individuals with high income and education level have shown to be connected more with social media for the preventive measures [29,30,31]. Furthermore, numerous studies argued that aged people followed better precautions by wearing a mask, using sanitizer, and keeping healthy respiratory hygiene [32,33]. Likewise, the relationships between the social determinants and prevention behaviors have presented that females [34], individuals with high literacy [29], and aged people [35] preferred to stay at home instead of visiting public places during an infectious period. However, according to the research conducted in the UK during a swine flu outbreak, individuals who have a low literacy rate/income level or are unemployed have avoided using public transport and visiting crowded places, in comparison with those individuals who have a high level of social determinants [33]. So, we can formulate following hypotheses.

**Hypothesis** **6** **(H6).** *There is no significant relationship between high-income individuals and preventive behaviors among people about COVID-19*.

**Hypothesis** **7** **(H7).** *There is no significant relationship between aged individuals and preventive behaviors among people about COVID-19*.

**Hypothesis** **8** **(H8).** *There is no significant relationship between gender of individuals and preventive behaviors among people about COVID-19*.

**Hypothesis** **9** **(H9).** *There is no significant relationship between high-educational individuals and preventive behavior among people about COVID-19*.

## 3. Materials and Methodology 

Research methodology, the principles and techniques used for gathering and analyzing data, plays an essential role in achieving the objectives of the study. This section presents the overall data sampling, research design, and data collection method used to find the objectives of the current study. An online survey was conducted by researchers in March 2020 during the COVID-19 outbreak using social media tools like Facebook, Twitter, WhatsApp, and Email. A link was developed, and the structured survey was shared with participants on this link through different social media tools like Facebook, Twitter, WhatsApp, and Email. The intention behind the selection of online data collection using social media tools was maintaining the social distancing principle. This research is based on individuals from different geographical areas of Punjab and Azad Jammu and Kashmir, Pakistan. The study was conducted on social media during 5 March 2020 to 25 March 2020. The sample size of 500 respondents was used through a random sampling method and examined with SPSS AMOS. The main reason for choosing this sampling method was that the researcher placed the questionnaire online. The researcher used a Likert scale of 5 points. The hypotheses were measured using a scale by [36].

### 3.1. Socio-Economic Characteristics of Respondents

Social determinants are considered very important in social science research and these were measured to check the significant direct effect of these control variables on preventive behavior among people about COVID-19. The essential statistical components are age, gender, education, and income. These demographic components were necessary for the assessment of our objectives. The details of these variables are given in Table 1.

### 3.2. Model of the Study 

**Figure 1:**
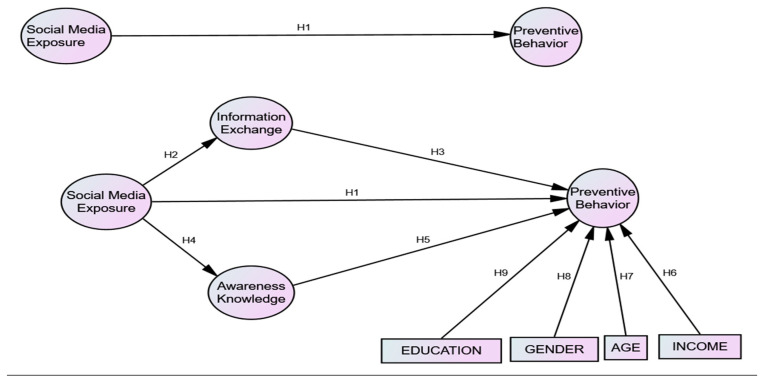
Conceptual Framework.

The proposed model and variables investigated in this study are demonstrated in Figure 1. 

## 4. Results and Discussions 

### 4.1. Structure Model

SEM technique was performed to examine the hypotheses discussed above. Table 2, Table 3 and Table 4 show the key consequences for the hypotheses. Researchers used path models to check the impact of social media on mediating variables, that is, information exchange and awareness knowledge regarding preventive behavior among people about COVID-19 as a dependent variable and checked the direct effect of control variables on preventive behavior among people about COVID-19. Additionally, path analysis and maximum likelihood method were used to verify the mediated impact of health communication (awareness knowledge and information exchange) among social media and preventive behavior. AMOS version 24 was used to check the statistical relationship between variables. 

Initially, we tested the model fit index with comparative fit index (CFI) and root mean square error of approximation (RMSEA); a CFI ≥0.97 and RMSEA ≤0.055 mean the fit was acceptable (Hu and Bentler, 1999). The indirect effect of social media on behaviors was calculated using the same statistical tool through 2000 bootstrap samples. Critical factor analysis (CFA) was used to test the discriminant and convergent validity of every construct of the measurement model. We also checked the factor score of each item, and all items exceeded the threshold of 0.6 (*p* < 0.001). The value of AVE ranged from 0.55 to 0.79 (all values are exceeding the threshold 0.5), and CR ranged from 0.82 to 0.92 (all exceeding the threshold of 0.7). 

According to the parameter estimation results of Table 4, the direct impact of social media exposure on preventive behaviors concerning H1 (β = −0.097 *p* < 0.001) showed an insignificant direct relationship between independent variable and dependent variable. Therefore, H1 was not supported. According to the results of H4 (β = 0.389, *p* < 0.01) and H2 (β = 0.377, *p* < 0.01), both showed significant direct effect of social media on awareness knowledge and information exchange. So, we accepted these two hypotheses. Therefore, we can say that social networking sites have been used as an important strategy to spread awareness and information at the time of emergency to control the COVID-19 outbreak. 

Health communications via social media were positively significantly influenced by awareness and information exchange and indirectly influenced the adoption of preventive healthcare behavior. H6, H7, H8, and H9 tested whether age, gender, income, and education would be insignificantly associated with preventive behaviors. The parameter estimates showed that H9 education (β = 0.106, *p* < 0.01), H7 age (β = −0.052, *p* < 0.01), H8 gender (β = 0.041, *p* < 0.01), and H6 income (β = 0.023, *p* < 0.01) have negatively insignificant relationships with preventive behaviors. All these control variables were supported according to our theory. It is not necessarily individuals with high literacy/income and aged people who avoid using public transport and crowded places. The effects of high social components were directly insignificant on preventive behavior to control the epidemic disease of COVID-19. According to the study findings, every type of individual can acquire an advantage through social media campaigns regarding the preventive behavior against COVID-19. 

### 4.2. The Direct and Indirect Effect of Mediating Variables on Preventive Behavior 

**Figure 2:**
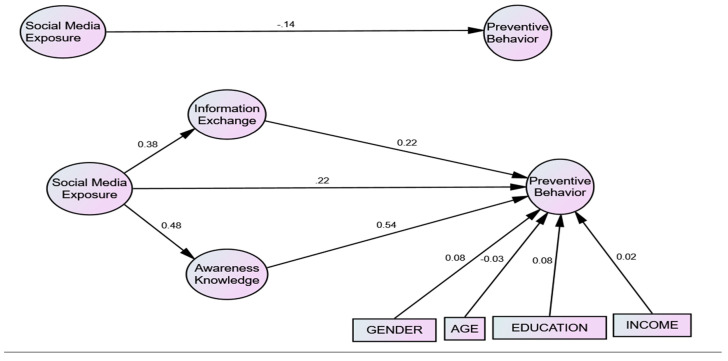
Mediating relationships.

H5 and H3 tested whether awareness knowledge and information exchange directly influenced preventive behavior during an infectious disease outbreak like COVID-19. Estimated parameters in Table 2 illustrated that awareness knowledge (β = 0.454, *p* < 0.001) and information exchange (β = 0.199, *p* < 0.001) have a positive significant direct relation with preventive behavior and have a full mediating effect between the social media and preventive behavior, as illustrated in Table 2 and Table 3 and Figure 2. Social media provides the possibility for individuals to be aware of private or public awareness campaigns. Eke [37] supported this theory that public awareness affects an individual behavior during an infectious disease outbreak to control its spread. Our study showed that public or private awareness through social media could overcome the spread of infectious disease.

The connectivity between the constructed hypotheses of our theory test is shown in Table 4, Table 5 and Figure 2. According to the results of direct relation, no direct relationship exists between social media exposure and preventive behavior, however, awareness knowledge and information exchange create a mediating effect between the social media exposure and preventive behavior, so there exists a strong relationship between social media exposure and preventive behavior with the full mediation of awareness knowledge and information exchange. 

## 5. Conclusions 

In conclusion, the COVID-19 outbreak in China significantly damaged the human population across the globe. This included widespread distrust, a high number of deaths, high public stress, and economic damage. This study analyzed the effect of social media on preventive behavior during the COVID-19 outbreak in Pakistan. Firstly, it should be counted that social media has become an increasingly popular source of awareness and information for health communications, especially during an outbreak. The data have been collected and analyzed as the outbreak started in Pakistan in 2020. 

This study examined how social media plays an essential role in formulating preventive behavior during the COVID-19 outbreak in Pakistan. The results of this research revealed that social media exposure is associated with two relevant variables, awareness knowledge and information exchange, and these variables mediate the relationship between social media exposure and preventive behavior among people regarding COVID-19. Social media reinforces and enhances health-related communication by raising awareness campaigns and disseminating reliable information to the users in an emergency regarding preventive behaviors. 

Social media has become a source of rapid information and can be updated promptly. If the utilization of social media becomes more accurate or scientific then the social media can provide a very efficient and user-friendly way of monitoring the facts and figures of epidemic both locally and at an international level. The use of social media as a communicating tool during the infectious disease outbreak is a new method of observation, but provides a potential source of an accurate and quick assessment of progression of the current condition of disease within communities. Social media has also become the most accessible and valuable tool, particularly in a social-economic and climatic context [36]. 

Mostly, developing nations like Pakistan do not have any excess to maintain and control the surveillance system in a timely manner during an infectious disease outbreak. Therefore, due to lack of resources, most developing nations use social media networks for health communication tools to prevent and control the spread of infectious disease in a community [37]. Thus, social media can afford a fast method of surveillance that forecasts the real-time burden of infectious disease and hence also can guide preventive strategies to control the epidemic.

The study has some limitations as only data from Pakistan were collected. Therefore, the results may not be easily generalized to other developing countries, but are useful for politicians, health administrators, governments, policymakers, and social scientists, especially for those whose circumstances are like those in Pakistan. The conceptual structural equation model provides useful information for policymakers and practitioners to enhance preventive behavior through the adoption of appropriate awareness strategies and information exchange and social media policies. The study demonstrates how social media exposure indirectly impacts preventive behavior and illustrates the paths of influence through either awareness or information exchange. To the best of our knowledge, the study is probably the first in the concerned area. The study investigated how only some social variables can help prevent COVID-19. Future researchers can investigate other variables lying under the category of social sciences and their role in dealing with COVID-19 and its impacts. The future studies can also specify the sectors, like health workers, education, police, and other security agencies.

## Figures and Tables

**Table 1:** Demographic Analysis.

Variable	Category	Frequency	Percentage
Gender	Male	361	72.2
	Female	139	27.8
Age (Years)	20–30	79	15.8
	31–40	150	30.0
	41–50	100	20.0
	51–60	80	16.0
	61 and above	91	18.2
Qualification	Bachelors	114	22.8
	Masters	192	38.4
	Postgraduates	132	26.4
	Diplomas	51	10.2
	Others	11	2.2
Income	1–10,000	48	9.6
	10,000–20,000	99	19.8
	20,000–30,000	165	33.0
	30,000–40,000	113	22.6
	40,000–above	75	15.0

**Table 2:** SEM Analysis.

Contract	Item	Loading	Mean	SD	AVE	CR
**Social Media Exposure**	SC1	0.65	3.57	0.76	0.55	0.82
	SC2	0.73				
	SC3	0.69				
	SC4	0.75				
**Awareness Knowledge**	AW1	0.74	3.54	0.79	0.62	0.89
	AW2	0.66				
	AW3	0.75				
**Information Exchange**	IF1	0.72	3.40	0.84	0.63	0.83
	IF2	0.74				
	IF3	0.68				
	IF4	0.71				
**Preventive Behavior**	PB1	0.79	3.55	0.86	0.79	0.92
	PB2	0.78				
	PB3	0.61				

**Table 3:** Overall fit index of the CFA model.

Fit Index	Score	Recommended Threshold Value
Absolute fit measures		
CMIN/df	1.787	≤2 ^a^; ≤ 5 ^b^
GFI	0.952	≥0.90 ^a^; ≥0.80 ^b^
RMSEA	0.040	≤0.8 ^a^; ≤0.10 ^b^
Incremental fit measures		
NFI	0.828	≥0.90 ^a^
AGFI	0.936	≥0.90 ^a^; ≥0.80 ^b^
CFI	0.914	≥0.90 ^a^
Parsimonious fit measures		
PGFI	0.071	The higher the better

a: Acceptability: Yes, acceptable; b: Acceptability: Marginal; Chi-square minimum/df (CMIN/df); goodness-of-fit index (GFI); root mean square error of approximation (RMSEA); normed-fit-index (NFI); adjusted-goodness-of-fit index (AGFI); comparative fit index (CFI); value and the parsimony-goodness-of-fit-index (PGFI)

**Table 4:** Regression weights (group number 1—Default model).

Hypothesis			Estimate	S.E.	C.R.	P	Effect	Results
Information Exchange	<--	Social exposure	0.377	0.093	5.306	***	**+**	Supported
Awareness Knowledge	<--	Social exposure	0.389	0.094	5.090	***	**+**	Supported
Preventive Behavior	<--	Social exposure	−0.097	0.118	−1.181	0.238	**-**	Not Supported
Preventive Behavior	<--	Information Exchange	0.199	0.079	2.781	0.005	**+**	Supported
Preventive Behavior	<--	Awareness Knowledge	0.454	0.108	4.956	***	**+**	Supported
Preventive Behavior	<--	Income	0.023	0.037	0.433	0.665	**−**	Supported
Preventive Behavior	<--	Education	0.106	0.042	2.028	0.043	**−**	Supported
Preventive Behavior	<--	Age	−0.052	0.032	−0.986	0.324	**−**	Supported
Preventive Behavior	<--	Gender	0.041	0.098	0.790	0.429	**−**	Supported

*** means relationships are significant at p-value 0.000; + means positive effect and – means negative effect

**Table 5:** Direct/indirect and total effects (group number 1—default model).

Predictor	Education	Age	Income	Gender	Social Exposure	Knowledge	Information Exchange
**Direct Effect**							
Awareness Knowledge	0.000	0.000	0.000	0.000	0.476	0.000	0.000
Information Exchange	0.000	0.000	0.000	0.000	0.492	0.000	0.000
Preventive Behavior	0.085	−0.032	0.016	0.077	−0.140	0.535	0.220
**Indirect Effect**							
Preventive Behavior					0.363		
**Total Effect**							
	0.085	−0.32	0.016	0.077	0.363 + (−0.140) = 0.223	0.535	0.220
